# The Effects of Rainfall on the Population Dynamics of an Endangered Aquatic Plant, *Schoenoplectus gemmifer* (Cyperaceae)

**DOI:** 10.1371/journal.pone.0157773

**Published:** 2016-06-21

**Authors:** Koshi Kitamura, Satoshi Kakishima, Takashi Uehara, Satoru Morita, Kei-ichi Tainaka, Jin Yoshimura

**Affiliations:** 1 Graduate School of Science and Technology, Shizuoka University, Hamamatsu, Shizuoka, Japan; 2 Department of Mathematical and Systems Engineering, Shizuoka University, Hamamatsu, Shizuoka, Japan; 3 Marine Biosystems Research Center, Chiba University, Kamogawa, Chiba, Japan; 4 Department of Environmental and Forest Biology, State University of New York College of Environmental Science and Forestry, Syracuse, New York, United States of America; Helmholtz Centre for Environmental Research - UFZ, GERMANY

## Abstract

The conservation of aquatic plants in river ecosystems should consider the wash-out (away) problem resulting from severe rainfall. The aquatic plant *Schoenoplectus gemmifer* is an endangered species endemic to Japan. Our previous study reported that the population size of *S*. *gemmifer* in Hamamatsu city, Japan, had decreased by one-tenth because many individuals had been washed out by a series of heavy rains in 2004. However, there is insufficient information on the ecological nature of this endangered aquatic plant for adequate conservation. In this paper, we report the population dynamics of one population in Hamamatsu city from 2004 to 2012 in relation to rainfall. We surveyed the number and growing location of all living individuals in the population 300 times during the study period. To examine the temporal changes of individual plants, we also counted the number of culms for 38 individuals in four observations among 300 records. Decreases and increases in the population size of this plant were associated with washing out and the settlement of gemmae (vegetative propagation), respectively. The major cause of the reduction in the population size was an increase in the number of washed-out individuals and not the decreased settlement of gemmae. The wash-out rates for small and large individuals were not significantly different. Small individuals having a stream form with linear leaves resisted flooding, and large individuals were often partially torn off by flooding events. Modification of river basins to reduce the flow velocity may be effective for the conservation of *S*. *gemmifer*.

## Introduction

The conservation of biodiversity is one of the most important environmental issues [1–4], and fresh water ecosystems are among the major targets of conservation biology [5–7]. Many species of aquatic organisms face extinction due to various anthropogenic pressures [8]. In many countries, aquatic plants are the most threatened groups of organisms [9]. The rapid reduction of aquatic plants is affected by habitat destruction, fragmentation, eutrophication, flooding, and drought [10–11]. Aquatic plants have evolved to adapt to the natural flow regimes of their habitats [10, 12]; however, river flow regimes are often modified by human activities, such as for flood control. It is unclear whether floods affect the populations of endangered aquatic plants in flood-controlled rivers. It is important to note that wash-out (away) is among the most common problems for aquatic plants in river ecosystems. Specifically, concrete basins are known to be highly susceptible to the washing out of aquatic plants in streams [13]. However, because washing out is a fundamental characteristic of aquatic plants in river ecosystems, we expect some degree of this phenomenon even in natural basins. It is important to examine the general features of wash-out incidents among endangered aquatic plants.

**Fig 1:**
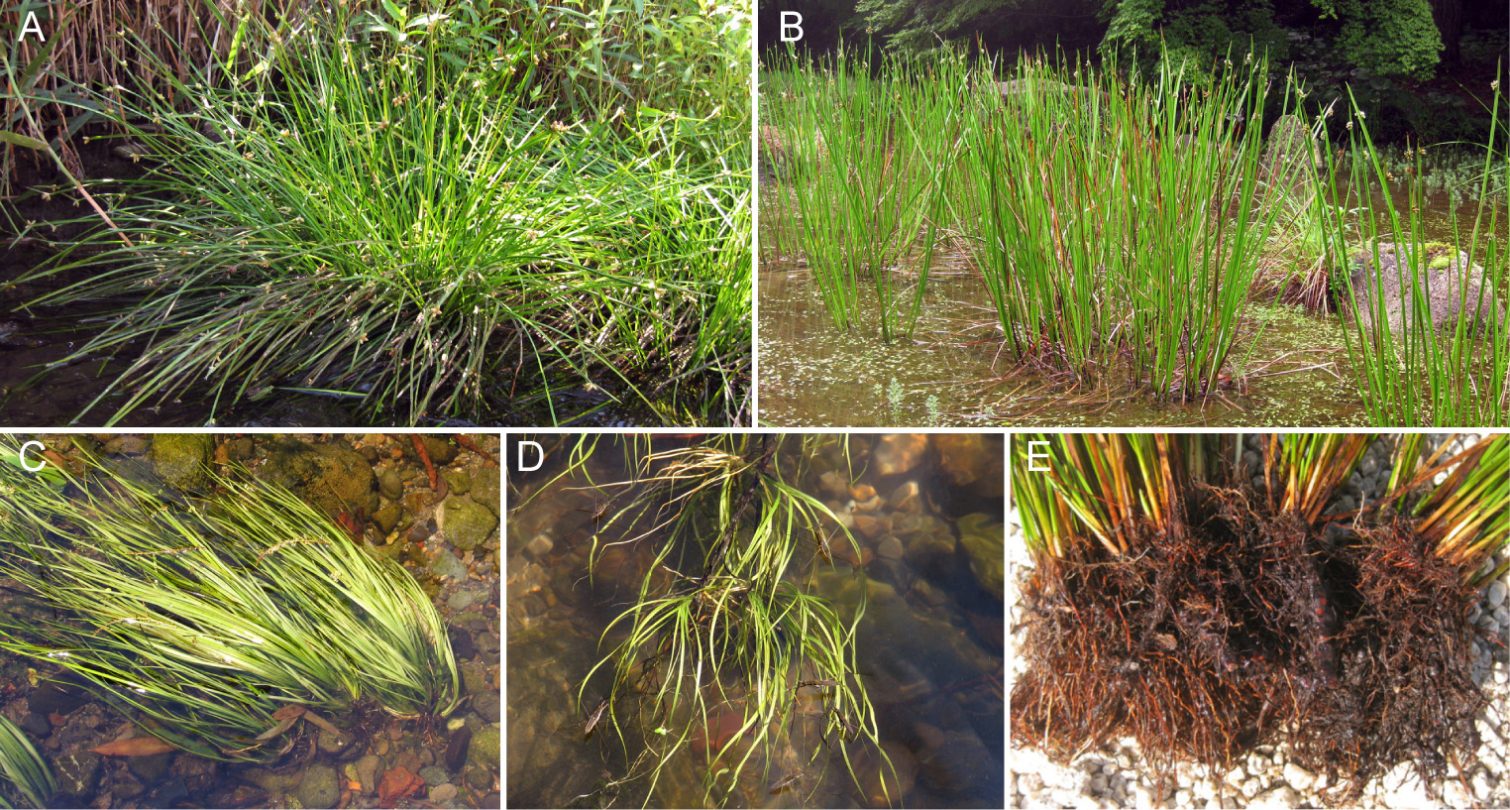
Morphologies of *S*. *gemmifer* and the closely related *S*. *triangulates*. (A) Gross morphology of *S*. *gemmifer*. Almost all large individuals become standing forms with flowers. (B) Gross morphology of *S*. *triangulates*. (C) A stream form of *S*. *gemmifer* with linear leaves. Almost all small individuals become stream forms. (D) The morphology of the settled gemmae of *S*. *gemmifer*. (E) The root system of a large individual that consists of several clumps of culms.

There have been many studies of the effects of flooding on riparian plants [10, 14–16]. However, detailed studies of endangered aquatic plants are rare. Aquatic plants growing in a river basin are expected to be adapted to river floods [10, 14]. It is important to understand how these plants have adapted or are protected from river floods or high water levels. In our previous study [17], we reported a survey of *Schoenoplectus gemmifer* C. Sato, T. Maeda & Uchino during 2004–2008 (Fig 1). This plant grows in rivers whose bottoms are covered by natural stones and gravel. The previous report [17] demonstrated that this plant is found in rivers or ponds with spring water (from the river bottom or nearby sources). Here, we report a follow-up and ongoing survey of this endangered aquatic plant. We focused on the wash-out and settlement of plants (or gemmae) on the natural river bottom. The population dynamics of *S*. *gemmifer* were surveyed from July 2004 to June 2012 by recording individual plants to examine the effects of precipitation on the reproduction (settlement of gemmae) and death (wash-out) of individual plants. We found that the survey period could be divided into two periods: (i) an increasing period in which the number of individuals slowly increased and (ii) a decreasing period in terms of the number of individuals. The aim of this study was to investigate how *S*. *gemmifer* maintains its population against floods by examination of population dynamics in both periods so that we can propose protection measures for this endangered species.

## Materials and Methods

### Species and survey site

*Schoenoplectus gemmifer* is an aquatic plant species endemic to Japan [18]. The genus *Schoenoplectus* (Rchb.) Palla (Cyperaceae) is widely distributed throughout the world and includes 77 species [19]. *Schoenoplectus gemmifer* (Fig 1A) is closely related to *S*. *triangulates* (Roxb.) Soják, an emergent plant characterized by densely tufted triangular culms (Fig 1B) that is common and widespread in Japan. Characteristic features of *S*. *gemmifer* include its linear stream-form leaves (Fig 1C) and the vegetative forms called gemmae (Fig 1D).

Because *S*. *gemmifer* grows in rivers with spring water only, its distribution is extremely limited, and its entire distribution includes approximately 20 locations [17]. We found out that some populations had already become extinct due to habitat modifications. Few plants were found in most populations, except for two large populations, one in Hamamatsu city, Shizuoka Prefecture, and the other in Oita Prefecture. Because all populations in Hamamatsu city are located in a residential area, their habitats are highly modified.

*Schoenoplectus gemmifer* can produce offspring by both asexual and sexual reproduction. The populations in Hamamatsu mainly reproduce asexually through vegetative forms called gemmae [17]. Gemmae are usually formed at or near the tip of culms (Fig 1D). The *S*. *gemmifer* populations in Hamamatsu were drastically reduced by heavy floods from July 2004 to March 2006 [17]. With consecutive heavy floods in the fall of 2004, a large number of individuals were washed out, and the population decreased to less than 10% of its initial size.

The reduction and increase in the abundance of this plant were associated with death (wash-out) and reproduction (settlement of gemmae). For the conservation of this species, a survey of the population dynamics of both wash-out and settlement processes is needed. Previous observations also indicated that the locations of the plants in a river tended to be crucial with respect to which plant was washed out [17].

We surveyed one population at the Higashikanda River (N 34° 45’, E 137° 41’), which is the largest population in Hamamatsu (see Kitamura et al, 2008 [17]). Both banks of the study site are covered by concrete walls, whereas the bottom is covered by natural stones and gravel. The river width is approximately six meters. The deepest location is approximately 15 cm on days without rainfall. The water depth increases by up to 100 cm or more during stormy days with consecutive heavy rains, although it rapidly returns to the usual level within a few days. Our observational study was conducted in a river on public land, and permission was not required to observe plants in rivers on public land in Japan.

### Examination of the population dynamics

From July 2004 to June 2012, 300 observations of the population at the Higashikanda River were conducted after heavy rains (more than 10 mm/day), as individuals are not washed out if the rainfall is less than 10 mm/day [17, 20]. The flow velocity in the study site is less than 0.4 m/s in a sprinkling rain of less than 10 mm/day. In these observations, we examined (i) the number of individuals, (ii) the growing locations of individuals, and (iii) the number of newly settled gemmae. The number of newly settled gemmae is related to asexual reproduction. Each individual was identified by its location, as the re-establishment of a washed-out individual was never observed [17].

### Examination of the number of culms

The number of culms per each individual was examined on four measurement dates: 18 May 2005 (denoted as Date Iothery function (PDF) with the observed), 19 August 2009 (Date II), 21 June 2011 (Date III) and 3 February 2012 (Date IV). The number of culms per individual is related to the biomass of the individuals. The population dynamics before Date I have been reported previously [17]. Prior to Date I, successive heavy rains caused by typhoons in the fall of 2004 had drastically decreased the population size.

### Rainfall monitoring

*Schoenoplectus gemmifer* was washed out by heavy rains of more than 10 mm/day [17]. The amount of rain (precipitation) is a critical factor for wash-out events, as it affects the flow velocity and river depth. Consecutive rains are suspected to be more critical, as they also considerably increase the depth and velocity of a river. Drastic reductions in population size were recorded immediately after the consecutive heavy rains in 2004 [17]. To clarify this influence, the relationship between the reduction of plants and both the amount of precipitation and the consecutiveness of rains were examined. The rainfall events of more than 10 mm/day and the consecutiveness of rains were monitored. Specifically, we verified whether the heavy rains stopped within a single day (single-day rains: SD) or continued for more than one day (continuous-days rains: CDs). We used the precipitation records of the Hamamatsu Weather Station (N. 34°42.5’, E. 137°43.1’), located approximately 4 km southeast of the study site. The precipitation records were obtained from the Japan Meteorological Agency [21].

### Statistical analyses

We examined the wash-out processes using statistical analyses. We proposed the following hypothesis: "both small and large individuals are evenly washed out". To test this hypothesis, we applied two types of statistical methods: Fisher's exact test and a Bayesian approach [22]. The former is inadequate to evaluate the evenness if a P-value is relatively large. The latter Bayesian method can be used to evaluate the evenness even if the sample size is relatively small. In the Bayesian method, we define *p*_*S*_(*t*) and *p*_*L*_(*t*) as the probability that small and large individuals survive at time *t*, respectively. Let $\bar{p}(t)$ be the geometric average $\bar{p}(t)=\sqrt{p_{L}(t)p_{S}(t)}$; then, obtain $p_{S}(t)={\bar{p}(t)}^{2(1-y)}$ and $p_{L}(t)={\bar{p}(t)}^{2y}$. These equations define a new parameter, *y*. If *y* = 1/2, then small and large individuals are equally washed out. If *y* > 1/2, a large individual is more likely to be washed out; when *y* = 1, only large individuals are washed out. By contrast, if *y* < 1/2, a small individual is more likely to be washed out, and when *y* = 0, only small individuals are washed out.

The relationship between the wash-out rate and the precipitation rate for each observation was analyzed by logistic regression using the R software [23]. The wash-out was described as a binary dependent variable (1: washed out; 0: survived), and precipitation was described by explanatory variables.

## Results

### Population dynamics

**Fig 2:**
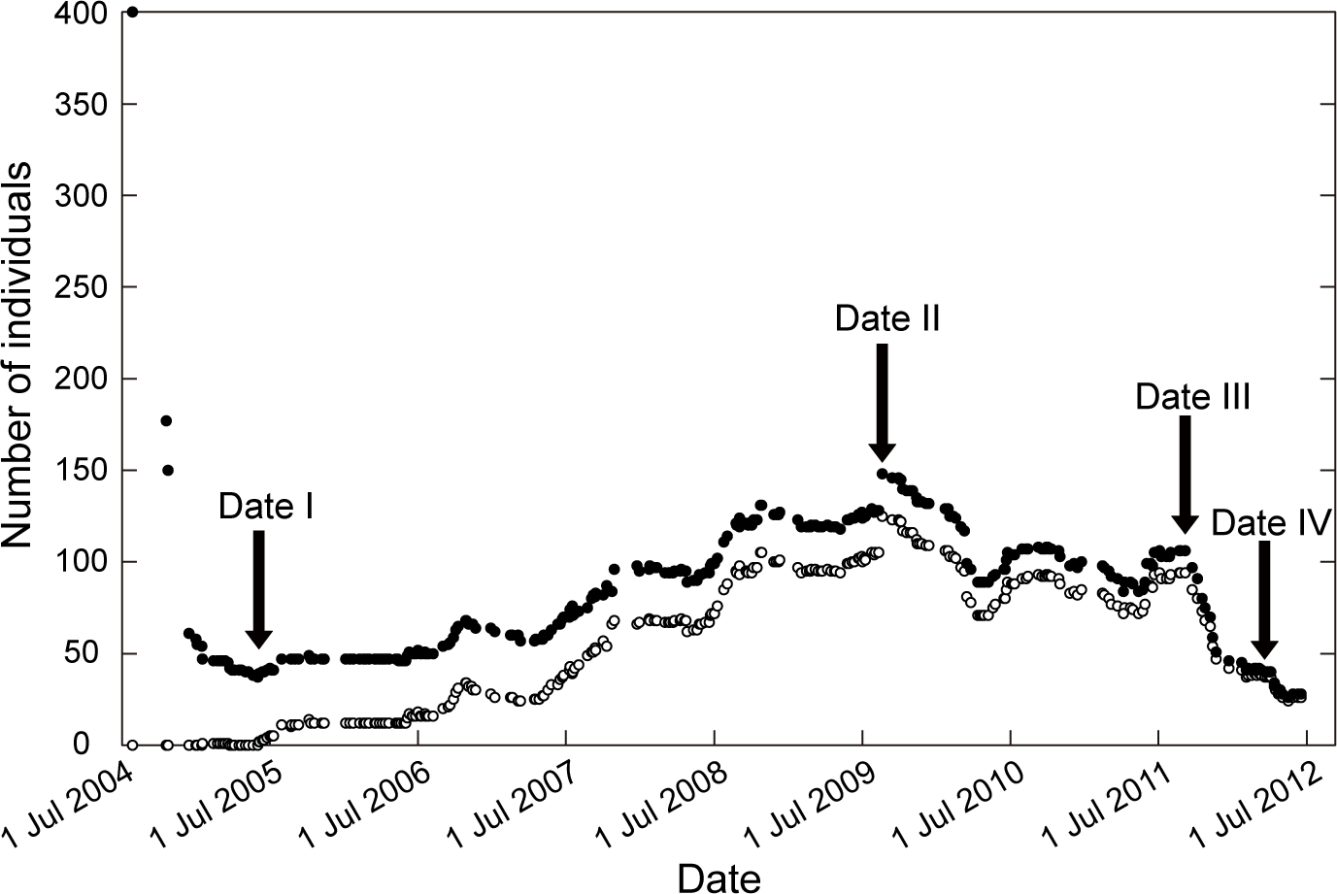
Population dynamics of *S*. *gemmifer*. The total population sizes of individuals (solid circles) and settled gemmae since February 2005 (open circles) are plotted from July 2004 to June 2012. Dates I–IV indicate the dates of culm measurements (Date I: 18 May 2005; Date II: 19 August 2009; Date III: 21 June 2011; and Date IV: 3 February 2012). Prior to Date I, the population size sharply decreased. The population size gradually increased during the period between Dates I–II and then decreased after Date II.

Three hundred observations were performed from July 2004 to June 2012 (S1 Table). The temporal dynamics of the population size (the total number of individuals) is illustrated in Fig 2. In 2004, the population size was rapidly decreased by severe floods. The population size was the smallest on Date I (40 individuals). After Date I, the population size gradually recovered until Date II (148 individuals). After Date II, the population size decreased again. The total number of individuals was 41 on Date IV. Thus, the temporal changes are roughly divided into two stages: (1) an “increasing period (stage)” for the period from Date I to Date II and (2) a “decreasing period (stage)” for the period after Date II. The total number of settled gemmae that were newly rooted after Date I was approximately proportional to the total population size (Fig 2). We should also note that all individuals were completely replaced by newly settled gemmae by the end of the observation period. After the observation period, *S*. *gemmifer* became extinct in October 2013. In total, 356 settled gemmae were washed out (S2 Table). The life span of these individuals was 435.0 ± 451.5 days (mean ± SD).

### Temporal change in the number of culms

In the increasing period, although the total number of culms per individuals increased, the average number of culms decreased; the average number of culms was 281.8 on Date I and 75.7 on Date II (Table 1 and S3 Table). In the decreasing period, both the total number of culms and the average number of culms decreased.

**Table 1:** Temporal changes in the population size, total number of culms, and mean number of culms per individual.

	Date I	Date II	Date III	Date IV
N	38	148	105	41
Total	10709	11204	6301	1636
Mean ± SD	281.8 ± 199.6	75.7 ± 67.4	60.0 ± 51.3	39.9 ± 29.3

Dates I–IV are defined in the text.

### Wash-out processes

Even during the increasing stage from Date I to Date II, the wash-out rate was high; among 38 individuals, 15 individuals were washed out (Table 2). Based on the number of culms, we classified all individuals as either small (less than 150 culms) or large (150 or more than 150 culms) individuals (S4 Table). From Date I to Date II, small individuals remained small (36.4%), grew and became large (27.3%), or were washed out (36.4%). Similarly, large individuals remained large (3.7%), were torn off and became small (55.6%), or were washed out (40.7%). Thus, large individuals consisting of several clumps of culms were easily torn off by floods and had a high probability of becoming small.

**Table 2:** Temporal changes with respect to individual size between Dates I–II.

	N	Large	Small	Washed
Large individuals	27	1 (3.7%)	15 (55.6%)	11 (40.7%)
Small individuals	11	3 (27.3%)	4 (36.4%)	4 (36.4%)
Total	38	4 (10.5%)	19 (50.0%)	15 (39.5%)

The plant sizes were categorized as follows: large individuals (≥150 culms) and small individuals (<150 culms). Large individuals become small when partly torn off by floods. Small individuals become large when they grow a sufficient number of culms. The loss by floods is indicated as washed (out). Parentheses indicate the percentage. Dates I–II are shown in the text.

**Fig 3:**
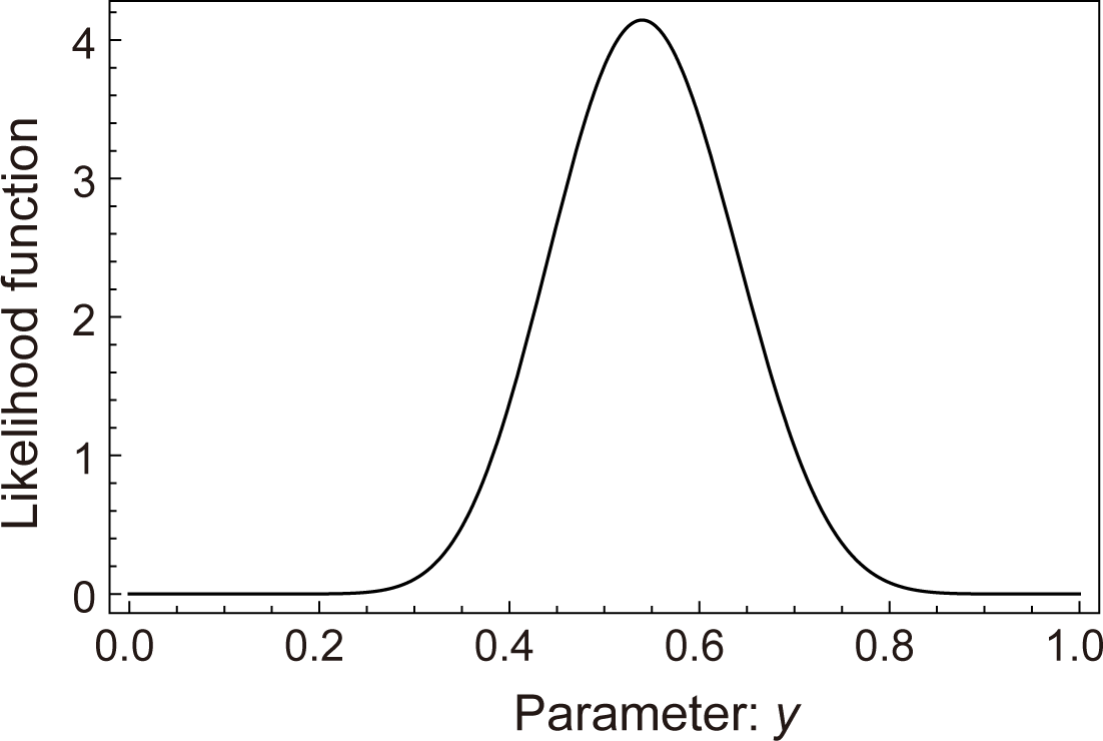
Result of the Bayesian inference. The likelihood function is depicted for the parameter *y*. The solid curve represents the marginal posterior distribution. We used the uniform distribution as a prior probability. The likelihood function was calculated from the data in Table 3.

The null hypothesis that “both small and large individuals were washed out evenly” was evaluated using two statistical tests. We calculated the expected number of surviving individuals on each measurement date (Table 3). Using the Fisher's exact test, we obtained *P* = 1.000 (df = 1), *P* = 0.817 (df = 1) and *P* = 0.494 (df = 1) for the intervals between Dates I and II, I and III, and I and IV, respectively. Therefore, we cannot reject the null hypothesis (i.e., there was no effect of plant size on the number of washed-out plants). We also performed Bayesian analysis to verify the hypothesis, as some sample sizes were too small (Table 3). The Bayesian approach showed a likelihood function against the parameter *y* (Fig 3) with a peak near *y* = 1/2. Therefore, we concluded that small and large individuals are evenly washed out.

**Table 3:** The observed and expected numbers of individuals on each measurement date.

		Date I	Date II	Date III	Date IV
Large individuals	Observed	27	16	9	2
	Expected		16.34	8.53	2.84
Small individuals	Observed	11	7	3	2
	Expected		6.66	3.47	1.16
Total	Observed	38	23	12	4

Surviving individuals were counted on each measurement date. Expected numbers were calculated assuming that there is no difference in wash-out rates between large and small individuals. Dates I–IV are defined in the text.

### Dynamics of the spatial distribution

**Fig 4:**
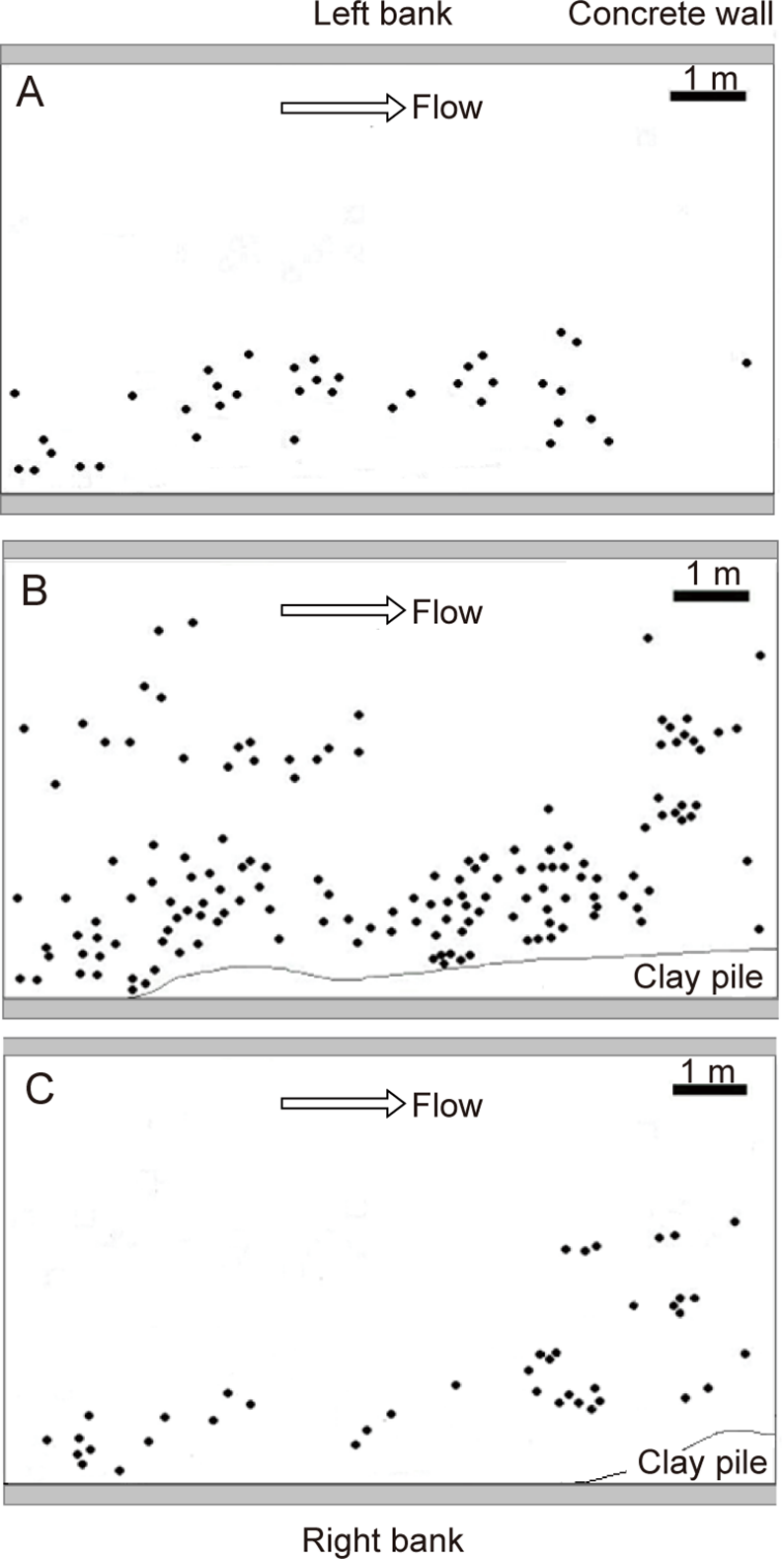
The spatial distribution at the study site. The positions of *S*. *gemmifer* are indicated by black dots. (A), (B) and (C) illustrate the spatial patterns on Dates I, II and IV, respectively. A pile of clay was present in (A), although its precise shape was not recorded. Dates I–IV are defined in the text.

Snapshots of the spatial distribution at the study site are shown in Fig 4 as the patterns determined on Dates I, II and IV. On Date I, all individuals were located near the right bank (Fig 4A) because all individuals except those in the right region were washed out in the fall of 2004 [17]. Note that the flow velocity is expected to be slowest near the right bank because the river bends gently upstream of the survey site [24]. This factor reasonably explains why almost all the individuals near the left (outer) bank had been washed out. On Date II, many individuals were located near the right bank, although some individuals were newly settled in the center or left region (Fig 4B). On Date IV, all of the remaining individuals were located in the center or right region (Fig 4C), as individuals that settled in the left region were washed out again. Note that the floods in the decreasing stage were less severe than in 2004.

We examined the spatial dynamics in terms of the settlement and wash-out. In both periods, the settlement of gemmae was highest in the right region and lowest in the left region (Table 4). The rates of settlement at the three positions were not significantly different between the two periods (Chi-square test: χ^2^ = 5.314, *P* = 0.07, df = 2). The number of settlements per day was also not largely different between the two periods (increase: 0.136; decrease: 0.114).

**Table 4:** Contingency table of the number of newly settled gemmae at different positions within the study site during periods I–II and II–IV.

	Left	Center	Right	Total
Period I–II	14	71	127	212
Period II–IV	1	32	69	102
Total	15	103	196	314

The surveyed site was divided into left, center and right regions. Period I–II is the increasing period, and period II–IV is the decreasing period. Dates I–IV are defined in the text.

In both periods, the wash-out rates of individuals were lowest in the right region and highest in the left region (Table 5). The wash-out rate in the decreasing period was significantly higher than in the increasing period (Chi-square test: χ^2^ = 98.201, *P* < 0.001, df = 1; Table 6). The number of washed-out individuals per day was distinctly different between the two periods (increase: 0.065; decrease: 0.233).

**Table 5:** Wash-out rates at different positions in the study site during the increasing and decreasing periods.

	Left	Center	Right	Total
Period I–II	57% (14)	45% (73)	37% (162)	41% (249)
Period II–IV	100% (6)	85% (71)	83% (173)	84% (250)
Total	70% (20)	65% (144)	61% (335)	62% (499)

Period I–II is the increasing period, and period II–IV is the decreasing period. Parentheses indicate the numbers of observed individuals. Dates I–IV are defined in the text.

**Table 6:** Contingency table of the number of washed-out and surviving individuals during the increasing and decreasing periods.

	Washed	Survived	Total
Period I–II	101	148	249
Period II–IV	209	41	250
Total	310	189	499

Period I–II is the increasing period, and period II–IV is the decreasing period. Dates I–IV are defined in the text.

### Effects of floods

**Fig 5:**
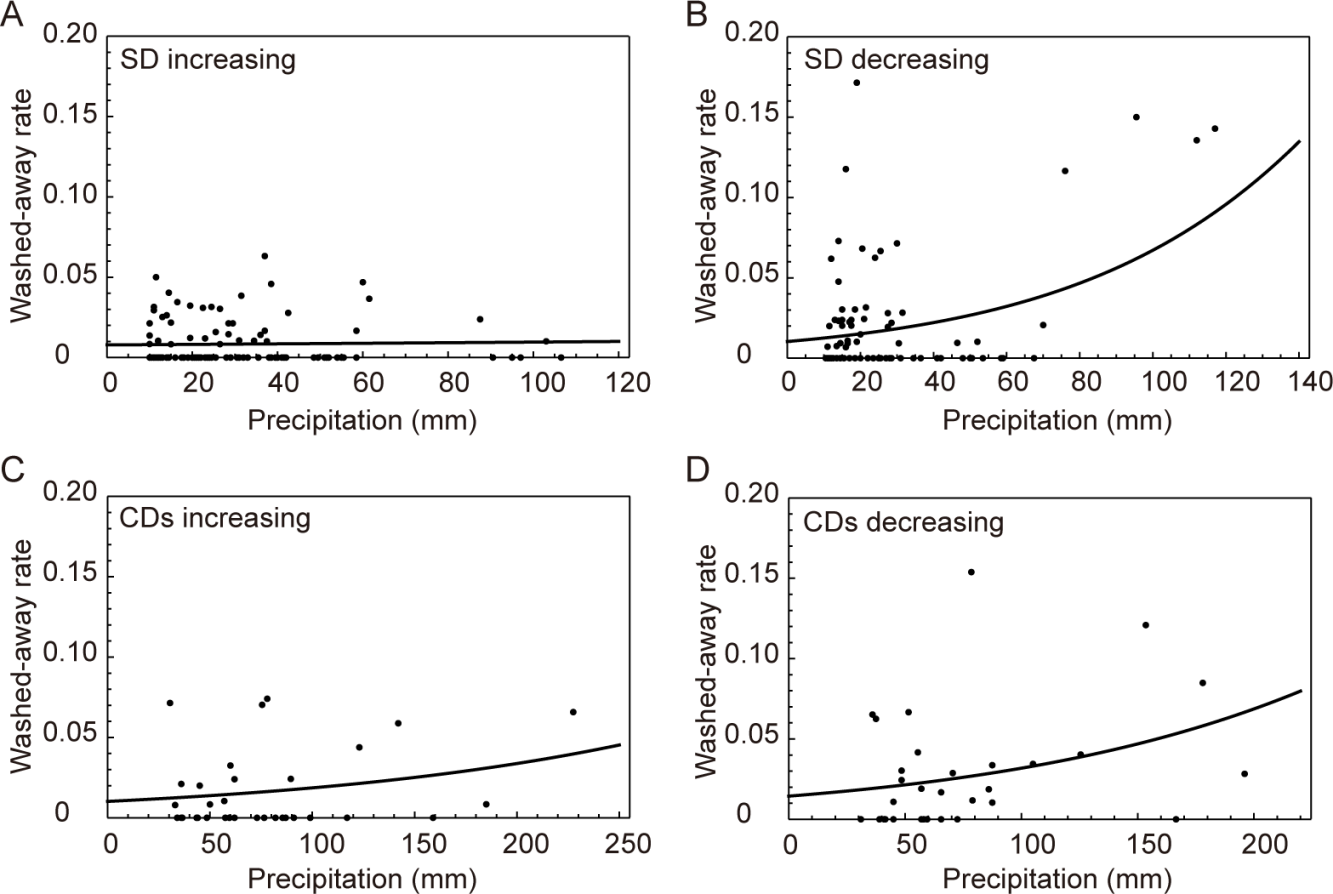
Relationships between the wash-out rates and precipitation during the increasing and decreasing periods. (A) Single-day rains (SD) in the increasing period (between Dates I and II). (B) SD in the decreasing period (after Date II). (C) The continuous-days rains (CDs) during the increasing period. (D) CDs during the decreasing period. SD denotes that the rain stopped in a single day, and CDs indicates that the rain continued over multiple days. Dates I–IV are defined in the text.

The wash-out rates of individuals are shown for two types of rainfall: SD (single-day rains) and CDs (continuous-days rains) (Fig 5). In the increasing period, the wash-out rates for SD did not significantly increase with increased precipitation (estimate ± SE = 0.002037 ± 0.005726, z-value = 0.356, *P* = 0.722). By contrast, the wash-out rates for CDs did significantly increase with increased precipitation (estimate ± SE = 0.006109 ± 0.002665, z-value = 2.292, *P* = 0.0219). However, during the decreasing period, the wash-out rates of both SD and CDs increased significantly with increased precipitation (SD: estimate ± SE = 0.019281 ± 0.003273, z-value = 5.89, *P* < 0.001; and CDs: estimate ± SE = 0.008086 ± 0.002156, z-value = 3.75, *P* < 0.001).

In the case of SD, the wash-out rates during the decreasing period were significantly higher than those in the increasing period (estimate ± SE = -0.84941 ± 0.14525, z value = -5.848, *P* < 0.001). Similarly, in the case of CDs, the wash-out rates in the decreasing period were also significantly higher than during the increasing period (estimate ± SE = -0.53766 ± 0.17976, z value = -2.991, *P* = 0.00278).

## Discussion

### Wash-out processes

*Schoenoplectus gemmifer* was frequently washed out by floods in the study site. Interestingly, there was no difference in the wash-out rates of large and small individuals (Table 3 and Fig 3). The persistence of small individuals during floods may be due to their shape [25,26]. Small individuals usually have a stream form with linear (narrow) leaves (Fig 1C). Linear leaves can persist during strong water flow [27, 28]. The stream form of small individuals seems to be effective against being washed-out. By contrast, large individuals have a standing form (Fig 1A). In addition to wash-out, large individuals were also frequently torn off partially by floods, becoming small individuals (Table 2). The partial loss of an individual may be related to the clump structures (Fig 1E); each individual consists of some subparts, which consist of a clump of culms. The clump structures may be effective in escaping from total loss [29].

### Dynamics of the spatial distribution

The spatial dynamics in the decreasing period from Date II to Date IV was opposite of that observed during the decreasing process in 2004 [17]. While individuals in the center region were washed out first in 2004, individuals in the left region were washed out first during the decreasing period (Fig 4C). This difference is suspected to be due to differences in the wash-out processes. While many plants were washed out almost simultaneously due to the severe consecutive rains in 2004, individuals were gradually washed out during the decreasing periods of 2009–2012. The flow velocity on ordinary days may be fastest near the left bank because of the gentle bend upstream, whereas the flow velocity during severe floods may be fastest in the center region.

There are several potential reasons that the recovery of the center region was faster than that of the left region (Fig 4B). The reason for low recovery in the left region was that the settlement of gemmae was smallest and the wash-out rates of individuals were highest (Table 4; Table 5). The new establishment of gemmae in the center region may be explained in two different ways. One explanation is that the settled gemmae originated from the right region, which can easily explain why the center region recovered faster than the left region. The other explanation is that the settled gemmae were derived from the habitat in the upper streams.

### Effects of floods

During the increasing period, CDs (continuous-days rains) significantly increased the wash-out rates, whereas SD (single-day rains) had no effect on the wash-out rates (Fig 5). By contrast, during the decreasing period, both SD and CDs significantly affected the wash-out rates. These results suggest that the decreasing period was caused by the increase in wash-out rates. The reason for the difference in the wash-out rates between SD and CDs may be due to the difference in the population structure: many plants were larger during the increasing period, whereas almost all plants were smaller in the decreasing period (Table 1). Generally, larger individuals are superior to smaller ones with respect to their survival rates [30–33]. Therefore, after Date II, the habitat environment in the study site might have become worse for *S*. *gemmifer* growth. Plausible environmental variables include changes in the light condition (shade) as affected by trees or bushes along the stream [32, 33], water quality such as nutrients and pollution, and the clarity and temperature of the water [13, 34, 35]. The causes of the changes in individual size of *S*. *gemmifer* at the study site should be examined in a future study.

## Conclusion

Herein, we discuss the conservation policy of this endangered species endemic to Japan. From the observations during 2004–2012, the overall population dynamics profile indicates that *S*. *gemmifer* is critically endangered in the Higashikanda River. This plant is at a high risk of extinction due to future heavy consecutive rains, such as those that occurred in 2004 [17]. This study indicates that any small activities that modify the river basin and reduce the flow velocity might be effective in reducing the number of washed-out plants. For example, placing a large stone or block in the stream might effectively protect the plants from wash-out [20].

Another *S*. *gemmifer* population in the upstream region of the Higashikanda River was recently destroyed by a flood-control project (S1 Fig). This endangered aquatic plant is not well recognized by the public. It is necessary to increase public recognition of this critically endangered species to stop careless flood control activities by governmental offices [36]. Increased recognition is also important to encourage the local government to act as an agency for conservation, as public requests are critically important for the government to take any conservation action [37]. We stress that the entire distribution of this species has been recorded only in approximately 20 locations [17]. Most habitats have already been destroyed. If we lose the Hamamatsu population, the Oita population would become the only remaining large population of this species in the world.

## Supporting Information

S1 Fig Habitat destruction by flood-control projects. (A) The habitat of *S*. *gemmifer* and *S*. *triangulates* before construction. The photo was taken at another site of the Higashikanda River on 7 June 2009. (B) Habitat of *S*. *gemmifer* and *S*. *triangulates* after construction. Both plants disappeared after the flood-control projects. The photo was taken at the same location on 10 September 2010.
(TIF)

S1 Table Temporal changes in the number of newly settled individuals and the total number of individuals. (XLS)

S2 Table The life span, settlement dates, and wash-out dates of settled gemmae. (XLSX)

S3 Table The number of culms of each individual on four measurement days. Dates I–IV are defined in the text.
(XLSX)

S4 Table Temporal changes in the number of culms of 38 individuals. The numeral "zero" indicates that the plant was washed out. Dates I–IV are defined in the text.
(XLSX)
