# Supplementary figures and images for: The Effects of Rainfall on the Population Dynamics of an Endangered Aquatic Plant, Schoenoplectus gemmifer (Cyperaceae)
Source: PLoS One. 2016 Jun 21;11(6):e0157773. doi: 10.1371/journal.pone.0157773 (PMC4915625; doi:10.1371/journal.pone.0157773)

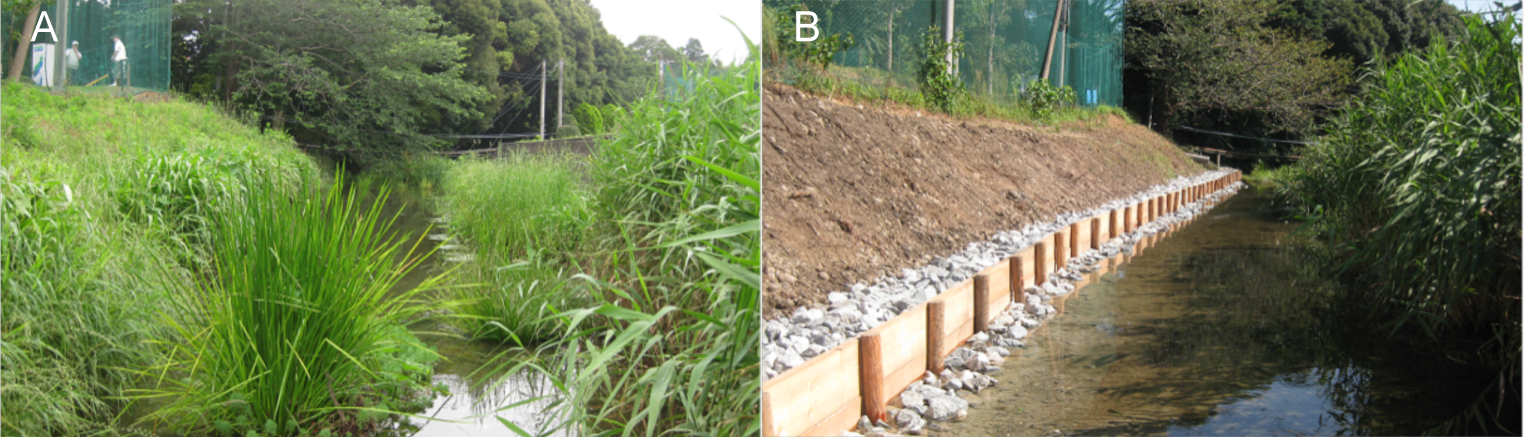

Supplement: S1 Fig — (A) The habitat of S. gemmifer and S. triangulates before construction. The photo was taken at another site of the Higashikanda River on 7 June 2009. (B) Habitat of S. gemmifer and S. triangulates after construction. Both plants disappeared after the flood-control projects. The photo was taken at the same location on 10 September 2010. (TIF) [file pone.0157773.s001.tif]
